# Supplementary material for: The Systems Biology Research Tool: evolvable open-source software
Source: BMC Syst Biol. 2008 Jun 29;2:55. doi: 10.1186/1752-0509-2-55 (PMC2446383; doi:10.1186/1752-0509-2-55)
Supplement: Additional file 1 — SBRT Archive. An archive of the current version of the Systems Biology Research Tool. [file 1752-0509-2-55-S1.zip › sbrt-1.4.0/doc/developers_guide/api/sbrt/kernel/math/geom/package-summary.html]

sbrt.kernel.math.geom


|  |  |  |
| --- | --- | --- |
| **Overview** \| **Package** \| Class \| **Use** \| **Tree** \| **Deprecated** \| **Index** \| **Help** | |  |
| **PREV PACKAGE**   **NEXT PACKAGE** | **FRAMES**    **NO FRAMES**     **All Classes** |  |


---

## Package sbrt.kernel.math.geom

Provides classes and interfaces for creating objects relevant to computational
geometry.

**See:**
  
          **Description**

| **Interface Summary** | |
| --- | --- |
| **ConvexPolytope<V>** | This interface is used to represent convex polytopes that are represented as the intersection of a finite set of half-spaces. |

| **Class Summary** | |
| --- | --- |
| **HashConvexPolytope<V>** | This class is a concrete implemenation of `ConvexPolytope`. |
| **HashConvexPolytopeOld<V>** | This class is a concrete implemenation of `ConvexPolytope`. |
| **ParallelHyperplanes<V>** | This class is used to represent a pair of bounding parallel hyperplanes. |

| **Exception Summary** | |
| --- | --- |
| **DimensionException** | This class is used to indicate invalid dimensions. |

## Package sbrt.kernel.math.geom Description

Provides classes and interfaces for creating objects relevant to computational
geometry.

---


|  |  |  |
| --- | --- | --- |
| **Overview** \| **Package** \| Class \| **Use** \| **Tree** \| **Deprecated** \| **Index** \| **Help** | |  |
| **PREV PACKAGE**   **NEXT PACKAGE** | **FRAMES**    **NO FRAMES**     **All Classes** |  |


---
